# GAP-43 is associated with faster amyloid-associated neurodegeneration and cognitive decline in Alzheimer’s disease

**DOI:** 10.3389/fneur.2025.1629389

**Published:** 2025-09-26

**Authors:** Yaxin Li, Xuanming Xu, Lian Tang, on behalf of the Alzheimer’s Disease Neuroimaging Initiative

**Affiliations:** ^1^Department of Laboratory Medicine, Nantong First People’s Hospital and The Second Affiliated Hospital of Nantong University, Medical School of Nantong University, Nantong, China; ^2^Department of Clinical Laboratory, Beijing Ditan Hospital, Capital Medical University, Beijing, China; ^3^Department of Neurology, Beijing Tiantan Hospital, Capital Medical University, Beijing, China

**Keywords:** Alzheimer’s disease, growth-associated protein 43 (GAP-43), neurodegeneration, cognition, Alzheimer’s Disease Neuroimaging Initiative (ADNI)

## Abstract

**Background:**

It has been proposed that amyloid-β (Aβ) deposition may trigger neurodegeneration and cognitive decline. The elevated levels of presynaptic growth-associated protein 43 (GAP-43) in cerebrospinal fluid (CSF) were significantly associated with Alzheimer’s disease (AD). To examine whether GAP-43 was associated with faster amyloid-associated neurodegeneration or cognitive decline, it was necessary to further explore whether Aβ deposition affected CSF GAP-43 through inflammation.

**Methods:**

A total of 671 participants from Alzheimer’s Disease Neuroimaging Initiative (ADNI) were enrolled with available baseline CSF GAP-43, microglia activation [measured by CSF soluble triggering receptor expressed on myeloid cells (sTREM2) and progranulin (PGRN)], neurodegeneration (measured by CSF t-tau), and Aβ pathology (measured by amyloid-PET). To compare CSF GAP-43 levels across different Aβ and clinical stages, the analysis of variance (ANOVA) and Bonferroni *post hoc* tests were conducted. Multiple linear regression models were used to explore the association of CSF GAP-43 with sTREM2, PGRN, amyloid-PET, p-tau, t-tau and cognitive measures at baseline. Moreover, mediation models with 10,000 bootstrapped iterations were performed to investigate whether CSF GAP-43 was related to accelerated amyloid-associated neurodegeneration, then further contribute to cognitive decline, and how Aβ deposition affected CSF GAP-43 leading to neurodegeneration.

**Results:**

Compared with the amyloid− (A−) group, CSF GAP-43 was significantly higher at baseline in the amyloid+ (A+) group. When stratified by diagnosis, similar results were observed in A+ cognitively normal (CN) and A+ mild cognitive impairment (MCI), compared with A−CN or A−MCI participants. We found that baseline of CSF GAP-43 was positively related to CSF sTREM2, PGRN, amyloid-PET and t-tau, whereas it was negatively associated with cognition. Besides, CSF GAP-43 mediated the faster progression of amyloid-associated neurodegeneration and cognitive decline. Furthermore, the mediation analysis revealed that CSF sTREM2/PGRN was related to CSF t-tau mediated by CSF GAP-43 in the A+ group.

**Conclusion:**

Our findings provided evidence that CSF GAP-43 was related to the accelerated amyloid-associated neurodegeneration, and further contributed to cognitive decline. We also demonstrated that Aβ deposition may act as a trigger for synaptic dysfunction by promoting inflammation.

## 1 Introduction

Alzheimer’s disease (AD) as the usual type of dementia, is characterized by the accumulation of amyloid-β (Aβ) plaques, aggregated hyperphosphorylated tau, neurodegeneration, synaptic degeneration, increased neuroinflammation, and cognitive decline (1, 2). It is widely recognized that increased Aβ may act as the trigger and promote the progression of AD. In particular, Aβ changes initiate AD through the activation of harmful cascade processes with the development and aggregation of hyperphosphorylated tau, ultimately leading to neurodegeneration and dementia (3, 4).

Synaptic damage is generally recognized as a core feature of AD, and is closely related to cognitive impairment (5). Previous studies have demonstrated that presynaptic proteins appeared to be more affected than postsynaptic proteins in AD (6, 7). Growth-associated protein-43 (GAP-43) is a presynaptic membrane protein marker, predominantly expressed in the entorhinal cortex and hippocampus, also involved in synaptogenesis in the adult brain (8, 9). Elevated levels of cerebrospinal fluid (CSF) GAP-43 were observed in AD patients, reflecting the dysfunction of the presynaptic membrane (10, 11). A previous study has reported that CSF GAP-43 is closely associated with tau pathology and neurodegeneration in AD patients (12). Meanwhile, both animal and human studies have also indicated that synaptic loss could precede neurodegeneration (13, 14). And there were no difference in CAP-43 levels between healthy group and A−T− group in former research (15). However, a recent evidence showed that CSF GAP-43 was correlated with Aβ deposition in AD patients (16). Concurrently, a cross-sectional study also found CSF GAP-43 levels were elevated in the A+T− group compared with A−T− group in cognitively normal individuals (17). With the increasing number and size of Aβ plaques, it might induce microglial dysfunction, triggering excessive pro-inflammatory responses that impair synaptic and neuronal function, potentially leading to cognitive decline (18, 19). These findings suggest that Aβ deposition and related inflammation may contribute critically to synaptic injury. Therefore, we hypothesized that Aβ deposition served as a trigger factor, leading to subsequent synaptic dysfunction and neurodegeneration, and eventually causing cognitive impairments.

A study indicates that dysregulation of the innate immune system may play a pivotal role in triggering the initial events of the amyloid cascade (20). And there is also evidence that reactive microglia is frequently colocalized with amyloid plaques in the AD brain, implying an interaction between microglia and this critical pathological hallmark of AD (21). In addition, emerging finding from both animal models and human brains study, indicates the synaptotoxic role of amyloid deposition and microglia-induced neuroinflammation (22, 23). It is well known that AD susceptibility gene—triggering receptor expressed on myeloid cell 2 (TREM2) is associated with microglia activation (24). This gene can undergo ectodomain shedding by ADAM proteases, generating a soluble fragment known as soluble TREM2 (sTREM2) (25). Since sTREM2 is detectable in CSF, it serves as a marker of microglia activation and neuroinflammation. Meanwhile, progranulin (PGRN) is a secreted protein which is predominantly expressed in microglia within the central nervous system. Recently, CSF PGRN has been proposed as a hallmark of microglia-mediated neuroinflammation (26). Herein, we employed CSF sTREM2 and PGRN as biomarkers of microglial activation. Given the critical role of microglial activation in the link between Aβ deposition and synaptic dysfunction, an important question arises as to whether Aβ deposition induces synaptic injury via microglia-mediated inflammation, ultimately leading to neurodegeneration.

The purpose of this study was to investigate whether the relationship between Aβ deposition and neurodegeneration was mediated by CSF GAP-43, eventually leading to cognitive impairment. We further aimed to explore whether Aβ deposition induced synaptic dysfunction via microglia-induced neuroinflammation. To address this, CSF biomarkers were employed to reflect synaptic dysfunction (GAP-43) and microglia activation (sTREM2, PGRN). Firstly, we examined the associations among CSF GAP43, microglia activation markers (sTREM2, PGRN), amyloid-PET, CSF p-tau, t-tau and cognitive measurements. Then, a mediation model was used to study whether CSF GAP-43 mediated the relationship between amyloid-PET and CSF t-tau. Finally, we studied the role of microglia-induced neuroinflammation in this pathway. Our underlying hypothesis was that CSF GAP-43 was positively related to microglia activation, Aβ deposition, neurodegeneration and cognitive decline. Simultaneously, the relationship between CSF GAP-43 and CSF sTREM2/PGRN in microglia-mediated synaptic dysfunction, may play a crucial role in participants with abnormal Aβ levels, leading to subsequent neurodegeneration.

## 2 Methods

### 2.1 Study design

All data for this article were obtained from the Alzheimer’s Disease Neuroimaging Initiative (ADNI) database in January 2023. ADNI is a public–private project launched in 2003 and led by principal investigator Michael W. Weiner, MD. The primary aim of ADNI is to examine whether imaging, biochemical biomarker, genetic and clinical assessment can be combined for early determination and tracking of AD. And the inclusion criteria could be available in ADNI website.^1^ The ADNI study was conducted according to the Declaration of Helsinki. Meanwhile, the institutional review boards of all participating centers approved the procedures of the ADNI study. Furthermore, all participants provided written informed consent. As for up-to-date information, researchers can refer to the ADNI website.

### 2.2 Participants

A total of 671 participants were enrolled from the ADNI database with available baseline CSF GAP-43, CSF sTREM2, PGRN, t-tau, amyloid-PET, cognitive measurements as well as basic clinical characteristics. The ADNI investigators classified clinical status as cognitively normal CN: (Mini-Mental State Examination) MMSE >24, (Clinical Dementia Rating) CDR = 0, mild cognitive impairment (MCI: MMSE>24, CDR = 0.5) or AD dementia following former criteria (27). Moreover, participants with subjective memory complaints at baseline were included as the CN group in this study. Aβ status was determined based on the previously established cut-off value of 1.11 for Florbetapir (28). Participants were categorized into the A− group (amyloid-PET <1.11), and A+ group (amyloid-PET ≥1.11). And we combined Aβ status with clinical diagnosis to classify the participants. There were six groups were obtained: “A−CN,” “A+CN,” “A+MCI,” “A−MCI,” “A−AD” and “A+AD.”

### 2.3 CSF biomarkers

CSF samples were obtained via lumbar puncture, following the standardized protocol outlined in the ADNI procedures manual (see text footnote 1). The concentration of CSF p-tau and t-tau were assessed by the team at the University of Pennsylvania. The fully automated Roche Elecsys with Innogenetics (INNO-BIA AlzBio3; Ghent, Belgium) immunoassay kit-based reagents were used to analyze CSF core biomarkers (29). For the CSF sTREM2 and CSF PGRN measurements, a previously described enzyme-linked immunoassay (ELISA) approach was employed (26). The values of CSF sTREM2 were corrected based on the values of 4 internal standards (ISs). And the values of CSF PGRN were corrected based on inter-batch variation. Corrected values of sTREM2 and PGRN can be found under the name “MSD_sTREM2CORRECTED” and “MSD_PGRNCORRECTED” in the ADNI database. CSF GAP-43 was detected by an in-house ELISA at the Sahlgrenska University Hospital. And the details of ELISA procedure have been described previously (10). In brief, the procedure employed a combination of the monoclonal GAP-43 antibody NM4 and the polyclonal GAP-43 antibody ABB-135 to specifically detect GAP-43 with ranging from 312 to 20,000 pg/mL.

### 2.4 Amyloid-β PET

Following standardized protocols, all ADNI site performed 18F Flortaucipir PET, and amyloid-PET was acquired 50–70 min (4 × 5 min frames) after 18F-florbetapir injection. A more detailed description of amyloid-PET acquisition and analysis has been provided elsewhere (see text footnote 1). A COMPOSITE standardized uptake value ratio (SUVR) was calculated by regional Florbetapir to the mean uptake of the whole cerebellum. A cortical summary SUVR was figured by a composite cortical region, containing frontal, cingulate, parietal, and temporal areas (30). All data were obtained from the UCBERKELEYAV45_08_09_18.csv dataset.

### 2.5 Cognitive assessment

To reflect memory function and executive function, we applied ADNI composite executive function score (ADNI-EF) and memory function score (ADNI-MEM) measuring by psychometrically optimized approaches with items (31), and the detailed methods document applicable to this dataset was available from the ADNI database (see text footnote 1). The global cognition was reflected by CDR scores and Alzheimer Disease Assessment Scale (ADAS) 11, ADAS 13, ADAS delayed word recall (ADASQ4), the Mini-Mental State Examination (MMSE) (32). Notably, the scores of CDR, ADAS-11, ADAS-13, and ADAS Q4 are inversely related to cognitive performance; that is, higher scores on these scales indicate greater cognitive impairment. And higher scores of ADNI-EF, ADNI-MEM, and MMSE indicate better cognitive performance.

### 2.6 Statistical analysis

Kolmogorov–Smirnov test was employed to assess the normality distribution of CSF t-tau, amyloid-PET, CSF GAP-43, CSF sTREM2, PGRN and cognitive measurements. Abnormal distributions values were log-transformed. The log10-transforemed values were used for all statistical analyses conducted in this study. And continuous and categorical data were compared between different groups via *t*-tests and *χ*^2^-tests, respectively. To examine levels of CSF GAP-43 across different Aβ and clinical statuses, an analysis of covariance (ANCOVA) was used, followed by Bonferroni *post hoc* analysis. Subsequently, multiple linear regressions were applied to evaluate the association between CSF GAP-43 with CSF sTREM2, PGRN, amyloid-PET, CSF p-tau, t-tau and cognitive measurements, adjusting age, sex, education and *APOE ε4* status. Furthermore, three mediation models were employed in this study. First, we tested whether the associations between amyloid-PET and CSF t-tau were mediated by CSF GAP-43. The second mediation model was used to explore whether the association between amyloid–PET and cognitive measurements was mediated by CSF GAP-43 and CSF t-tau. The third mediation model was employed to explore whether CSF GAP-43 mediated the relationship between CSF sTREM2/PGRN and t-tau in the A+ group. This mediation analysis aimed to examine whether Aβ deposition can lead to synaptic injury through inflammation. All of those mediation analyses were performed with 10,000 bootstraps replications controlling for age, sex, *APOE ε4* status and education.

R software version 4.1.0 and IBM SPSS Statistics 23 were utilized for all the data analyses. The “lmer,” “lme4,” “car,” “mediate” and “ggplot2” in R version 4.1.0 software were employed to conduct analyses. For serial multiple mediators, we analyzed through the PROCESS Macro for SPSS developed by Hayes (33). And two-tailed *p* < 0.05 was set as the statistically significant.

## 3 Results

### 3.1 Participant characteristics

The detailed demographic, biomarker and clinical characteristics were displayed in Table 1. A total of 671 participants (310 A− group and 361 A+ group) were included. In this study, the mean age was 72.17 years old, with 45.16% female and 46.35% *APOE ε4* carrier. The cognitive decline in the A+ group was significantly higher than that of the A− group (MMSE, *p* < 0.001). Compared with the A− group, there was higher amyloid-PET (*p* < 0.001), p-tau (*p* < 0.001), t-tau (*p* < 0.001), and GAP-43 (*p* < 0.001) in the A + group. No significant differences were observed between the two groups in terms of education (*p* = 0.535), CSF sTREM2 (*p* = 0.251), or CSF PGRN (*p* = 0.794).

**Table 1:** Characteristics of participants based on group classification.

Characteristics	A− group	A+ group	*p*
*N*	310	361	
Age (mean ± SD, years)	70.81 ± 7.16	73.35 ± 7.33	0.598^a^
Gender (female/male)	135/175	167/194	0.535^b^
Education (mean ± SD, years)	16.47 ± 2.45	15.96 ± 2.69	0.364^a^
*APOE ε4* (*n*, %)	73 (23.55)	239 (66.20)	<0.001^b^
MMSE score (mean ± SD)	28.60 ± 1.78	26.66 ± 2.85	<0.001^a^
Amyloid-PET (centiloid)	1.01 ± 0.05	1.40 ± 0.17	<0.001^a^
CSF p-tau (pg/ml)	19.13 ± 8.05	33.79 ± 15.62	<0.001^a^
CSF t-tau (pg/ml)	216.75 ± 85.15	339.62 ± 143.31	<0.001^a^
CSF GAP-43 (pg/ml)	4504.44 ± 2327.76	6038.70 ± 3082.78	<0.001^a^
CSF sTREM2 (pg/ml)	3748.44 ± 2116.59	4091.40 ± 2188.04	0.251^a^
CSF PGRN (pg/ml)	1582.05 ± 376.64	1585.83 ± 447.00	0.794^a^

Categorical variables are presented as numbers and percentages. Continuous variables are presented as Means ± SDs. APOE, Apolipoprotein E; SD, Standard deviations; MMSE, Mini Mental State Examination; CSF, cerebrospinal fluid; t-tau, total-tau; GAP-43: Growth-associated protein-43; sTREM2: soluble triggering receptor expressed on myeloid cells 2; PGRN: progranulin.

a Comparison of subgroups were employed by Student’s *t*-tests.

b Comparison of subgroups were employed by *χ*^2^-tests.

### 3.2 Levels of CSF GAP-43 in different Aβ status and clinical statuses

**Figure 1:**
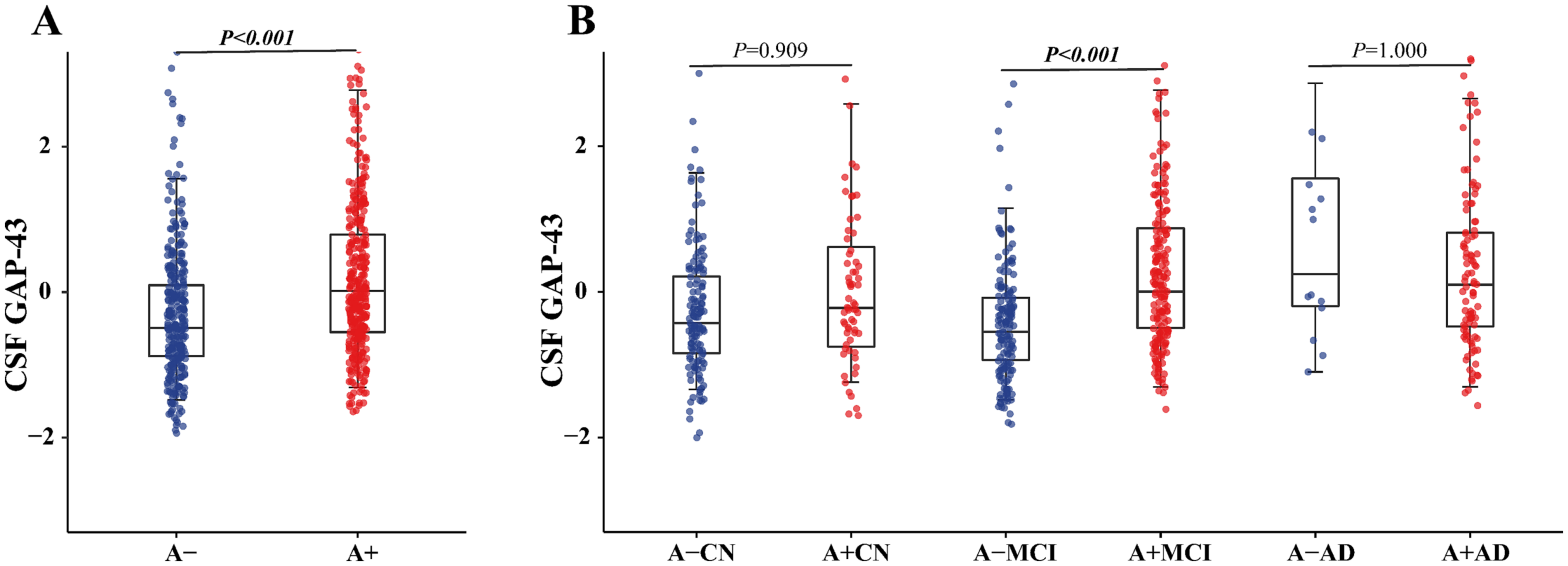
Scatterplots of CSF GAP-43 levels by Aβ status and clinical diagnosis. **(A)** Distribution of CSF GAP-43 in different Aβ groups, showing the A+ group with significant higher levels of CSF GAP-43 levels as compared with A− group (*p* < 0.001). **(B)** When stratified by clinical diagnosis, CSF GAP-43 levels were increased in A+CN (*p =* 0.909) and A+MCI (*p* < 0.001).

At baseline, our finding displayed CSF GAP-43 was obviously increased in the A + group compared with the A– group (*p* < 0.001, Figure 1A). As shown in Figure 1B, after combing with clinical staging, higher levels of CSF GAP-43 were seen in A+MCI compared A-MCI (*p* < 0.001). The levels of CSF GAP-43 also were observed an increase in A+CN compared with A–CN group (*p* = 0.909). In addition, there was no statistical differences of CSF GAP-43 in A+AD and A–AD group (*p* = 1.000).

### 3.3 Association of CSF GAP-43 with sTREM2/PGRN, Aβ depotion and neurodegeneration

**Figure 2:**
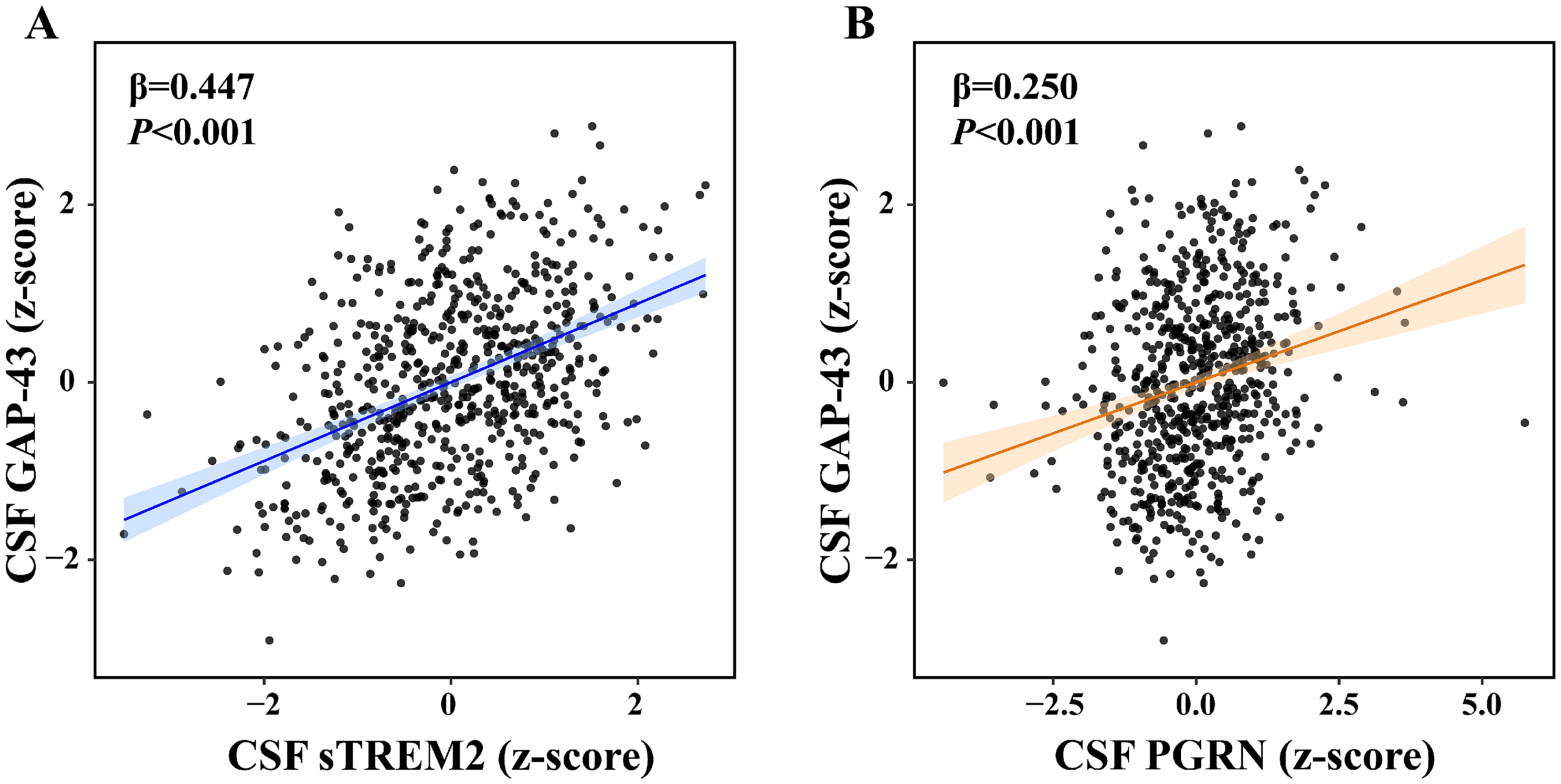
Scatterplots of the association between CSF GAP-43 with CSF sTREM2 and CSF PGRN. The scatterplots showed the relationships between CSF GAP-43 with CSF sTREM2 **(A)** and CSF PGRN **(B)** in the whole participants. The correlation analyses were performed using liner regression models. These plots exhibited that standardized beta-estimates (*β*) and *p*-values, which were adjusted for the effects of age, sex, *APOE ε4*, education and diagnosis.

**Figure 3:**
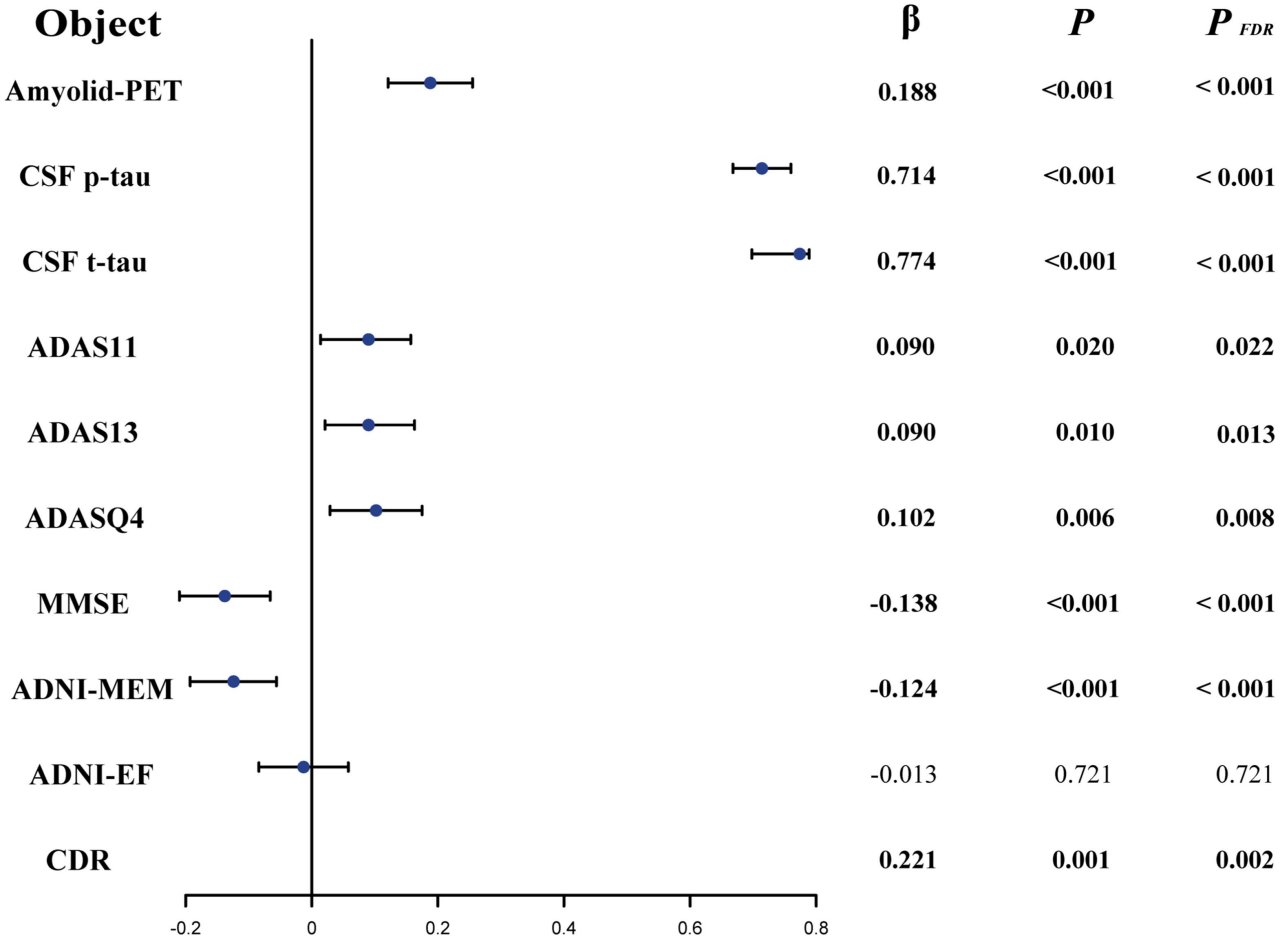
The relationship of between CSF GAP-43 with amyloid PET, CSF p-tau, CSF t-tau and cognitive measurements in the participants. Linear regression analyses were performed to examine the relationships, adjusting for potential confounding variables including age, education, sex, and *APOE ε4*. ADAS, Alzheimer Disease Assessment Scale; ADASQ4, ADAS delayed word recall; MMSE, Mini-Mental State Examination; ADNI-MEM, memory function score; ADNI-EF, ADNI composite executive function score.

Linear regression was performed to examine the relationships of CSF GAP-43 with CSF sTREM2/PGRN, Aβ pathology, and neurodegeneration across all participants (Supplementary Table S1). Amyloid-PET was regarded as a marker of Aβ pathology, while CSF t-tau served as a marker of neurodegeneration. We found the increased level of CSF GAP-43 was associated with higher level of CSF sTREM2 (*p* < 0.001, *β* = 0.447, Figure 2A), and PGRN (*p* < 0.001, *β* = 0.250, Figure 2B). As shown in Figure 3, individuals with higher levels CSF GAP-43 exhibited elevated amyloid-PET (*p* < 0.001, *β* = 0.188), as well as higher levels of CSF p-tau (*p* < 0.001, *β* = 0.714) and CSF t-tau (*p* < 0.001, *β* = 0.774) across the entire cohort.

### 3.4 Association of CSF GAP-43 with cognitive measurements

Next, the associations between CSF GAP-43 and cognitive measurements was studied. For the global cognitive scores, Figure 3 showed the increased levels of CSF GAP-43 were related to higher scores of ADAS11 (*p* = 0.020, *β* = 0.090), ADAS13 (*p* = 0.010, *β* = 0.090), ADASQ4 (*p* = 0.006, *β* = 0.102) and CDR (*p* = 0.001, *β* = 0.221) in the whole participants. The CSF GAP-43 level was negatively associated with MMSE (*p* < 0.001, *β* = −0.138). Regarding memory function, the CSF GAP-43 level was negatively correlated with ADNI-MEM scores (*p* < 0.001, *β* = −0.124). But there was no a significant relationship between CSF GAP-43 and ADNI-EF scores (*p* = 0.721, *β* = −0.013). After correction for multiple comparisons using the false discovery rate (FDR), the associations of amyloid PET, CSF p-tau, CSF t-tau and all cognitive measurements still remained statistically significant (*P_FDR_* < 0.05), except for ADNI-EF (*P_FDR_* = 0.721).

### 3.5 GAP-43 mediated the association between Aβ deposition and neurodegeneration

**Figure 4:**
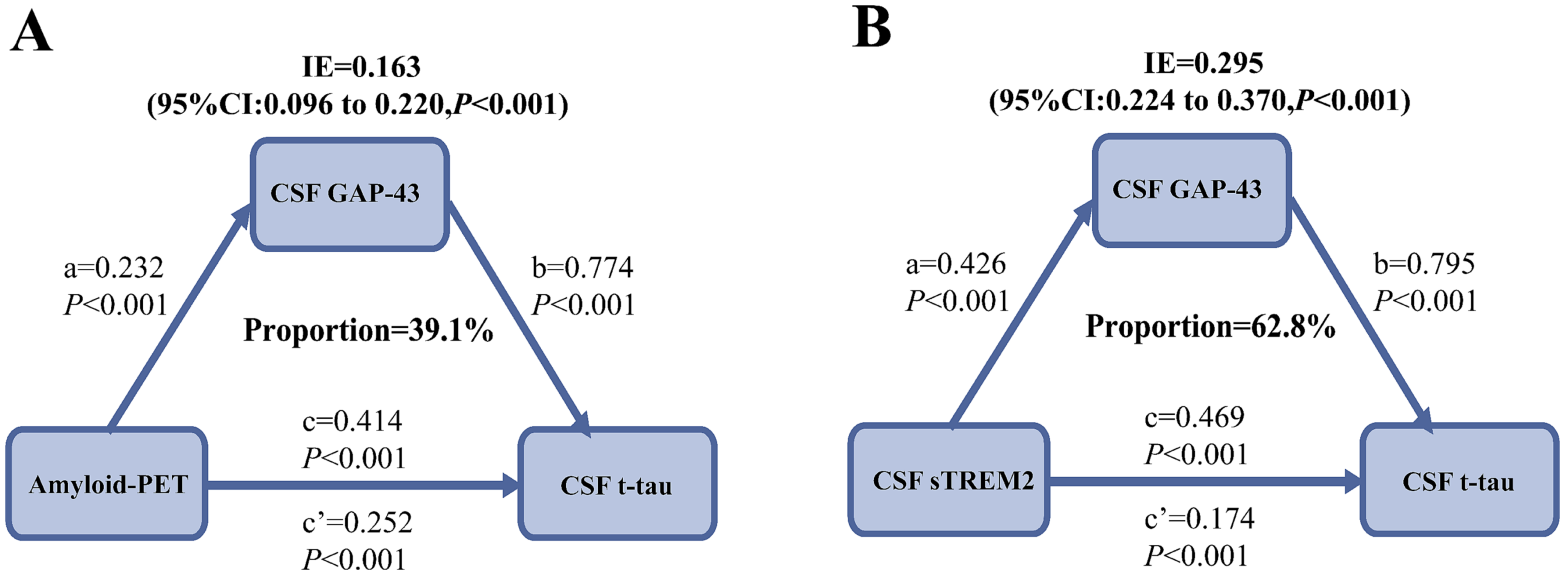
Mediation analyses with CSF t-tau as outcomes. **(A)** CSF GAP-43 mediated the relationship between amyloid-PET and CSF t-tau. **(B)** The relationship between CSF sTREM2 and CSF t-tau, was mediated by CSF GAP-43 in the A+ group. Beta estimates and *p* values for each path were displayed along the corresponding arrows. IE, indirect effect.

Moreover, we investigated whether CSF GAP-43 was associated with accelerated amyloid-related neurodegeneration. To this end, we performed the initial mediation pathway analysis in all participants: Amyloid-PET → CSF GAP-43 → CSF t-tau. Using bootstrapped mediation analyses with 10,000 iterations, we found the indirect effect of amyloid-PET on CSF t-tau via CSF GAP-43 was significant (*p* < 0.001, *β* = 0.163, 95%CI: 0.096 to 0.220, Figure 4A).

**Figure 5:**
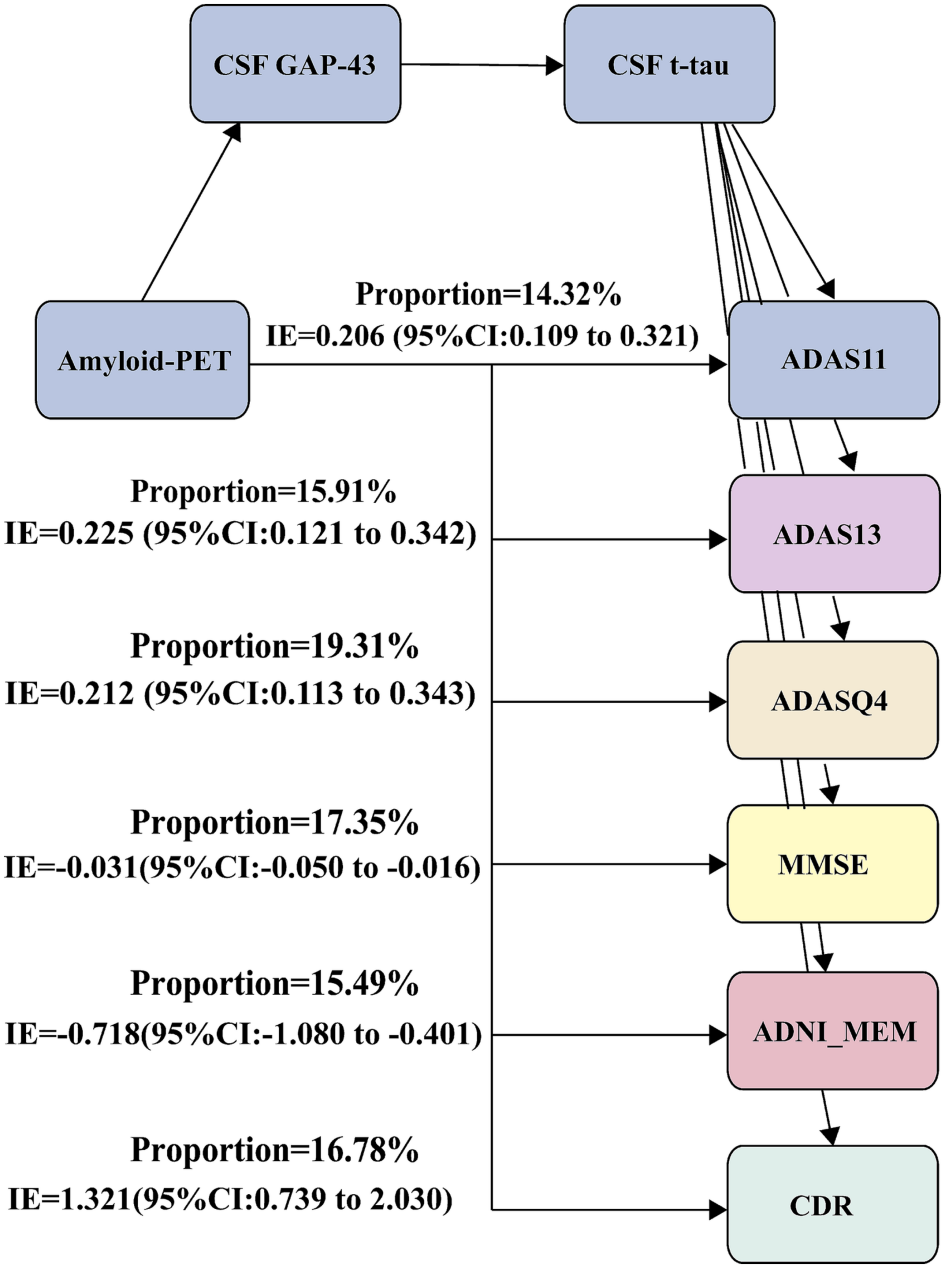
Mediation analyses with cognitive measurements as outcomes. Mediation analysis between amyloid-PET and cognitive measurements: Amyloid-PET → CSF GAP-43 → CSF t-tau → cognitive measurements. ADAS, Alzheimer Disease Assessment Scale; ADASQ4, ADAS delayed word recall; MMSE, Mini-Mental State Examination; ADNI-MEM, memory function score.

We further tested whether CSF GAP-43 and CSF t-tau contribute to the association between amyloid-PET and cognition. And the second mediation model was used: Amyloid-PET → CSF GAP-43 → CSF t-tau → ADAS11. The significant relationships were seen between amyloid-PET and ADAS11 (Figure 5). And mediation pathway revealed the effect of amyloid-PET with ADAS11 through CSF GAP-43 and CSF t-tau. For the sensitivity analysis, five different cognitive measurements were utilized to conduct mediation analysis, containing ADAS13, ADASQ4, MMSE, CDR, and ADNI-MEM. These obtained results were same as ADAS11 (Supplementary Table S2). Our data suggested that Aβ deposition maybe a key role in leading to synaptic dysfunction, ultimately causing neurodegeneration and cognitive impairment.

### 3.6 GAP-43 mediated the association between CSF sTREM2/PGRN and CSF t-tau at the A+ group

Considering the above findings, Aβ deposition appeared to play a crucial role in synaptic dysfunction. We further studied how Aβ deposition caused synaptic dysfunction, thereby leading to neurodegeneration. The third mediation model was used in the A+ group: CSF sTREM2/PGRN → CSF GAP-43 → CSF t-tau. The results indicated the significant indirect effects (*p* < 0.001, *β*_sTREM2_ = 0.295, 95%CI: 0.224 to 0.370, Figure 4B; *p* < 0.001, *β*_PGRN_ = 0.158, 95%CI: 0.083 to 0.240, Supplementary Figure S1). These results suggested that Aβ deposition may be associated with synaptic dysfunction via inflammation.

## 4 Discussion

Herein, we assessed whether CSF GAP-43 was associated with accelerated amyloid-related neurodegeneration, ultimately leading to cognitive decline. Furthermore, we investigated whether microglia-mediated inflammation played a critical role in Aβ-induced synaptic injury, which in turn led to neurodegeneration. Our data demonstrated that CSF GAP-43 was significantly increased in the A+ group compared to A− group and positively related to CSF t-tau, amyloid-PET, CSF sTREM2/PGRN, and cognitive decline at baseline. Mediation results revealed that CSF GAP-43 mediated the association of amyloid-PET with CSF t-tau, which eventually contributed to cognitive decline. Importantly, the mediation results further exhibited that the relationship between CSF sTREM2/PGRN and CSF t-tau was mediated by CSF GAP-43 in the A+ group. These results supported our hypothesis that the accumulation of Aβ deposition to a certain extent may injure synapses and result in neurodegeneration as well as cognitive impairment through microglia-mediated neuroinflammation in AD.

GAP-43 is essential for axonal growth and early synapse formation, highlighting its role in synaptogenesis. And neurogranin is mainly localized in the postsynaptic density and is linked to synaptic strength and calcium signaling. Moreover, SNAP-25 regulates presynaptic membrane fusion and neurotransmitter release (34). Neurogranin and SNAP-25 are more related to synaptic maturation and function. In this study, we used CSF GAP-43 as a biomarker to reflect early synaptic plasticity and axonal remodeling associated with neurodegenerative processes (35). We found that levels of CSF GAP-43 were increased at baseline in the A+ group compared to A− group. When stratified participants by Aβ status and clinical diagnosis, the same findings were obtained in the A+CN or A+MCI group compared with the A−CN or A+MCI group. Consistent with previous studies, increased levels of CSF GAP-43 were observed in the A+CN group compared with A−CN participants (36), as well as in A+MCI compared with the A–MCI group (10). There was no significant difference in CSF GAP-43 in the A+AD group (110 participants) compared with the A−AD group (13 participants). We suspected that the relatively small size of the A−AD group might have limited the statistical power during our analysis process, potentially affecting the reliability of the estimated differences of CSF GAP-43 levels in this subgroup. In addition, our results also showed CSF GAP-43 was positively related to amyloid-PET, CSF p-tau, and t-tau in the participants. Consistent with previous studies, CSF GAP-43 was related to Aβ deposition and tau pathology, occurring prior to neurodegeneration (10, 14, 16, 37). However, some previous studies reported that there was no obvious relationship between Aβ pathology and CSF GAP-43, which we suspect may be due to their use of CSF Aβ_42_ (15, 38). In our study, we employed amyloid-PET, which more accurately reflected Aβ deposition. Our data also demonstrated that CSF GAP-43 levels was higher in the A+ group and was associated with Aβ deposition, suggesting that Aβ deposition may trigger synaptic damage. However, the mechanism by which Aβ deposition influences CSF GAP-43 remains still unclear.

Our results also revealed that CSF GAP-43 was positively related to CSF sTREM2/PGRN in the participants. The microglia-synapse pathway has been identified as a key factor in AD progression, yet its underlying mechanism in AD remains unclear (19). Hence, several potential mechanisms may be as follows. One possible explanation was that the synapse was localized by classical complement proteins C1q and C3, which then mediated synapse loss by microglia (39). Another explanation was that microglia may release soluble synaptotoxic factors, ultimately promoting synaptic damage (40). Previous animal experiments had repeatedly shown that microglia activation was related to synaptic injury, which can eventually lead to neurodegeneration and cognitive decline (19). However, to our knowledge, there is lack of studies exploring the associations between microglia activation and synaptic dysfunction in human studies.

A study has suggested that Aβ deposition may be a mechanism to regulate synaptic activity (41). As Aβ pathology progresses, plaques began to deposit and increase, resulting in reduced glutamatergic transmission and synaptic damage in animal experiments (42, 43). Finally, this process might cause the detachment of tau from microtubules and the formation of tau tangles, which in turn contributed to neurodegeneration (44, 45). Some researches and reviews have showed that Aβ deposition initially leads to synaptic injury, which is subsequently followed by tau-related axonal degeneration (17, 46). Based on these research results, we found that CSF GAP-43 mediated the relationship between Aβ deposition and neurodegeneration, which eventually leading to cognitive impairment. And previous studies mainly focused on tau pathology in individuals (12). Our data provided evidence that GAP-43 was associated with faster amyloid-associated neurodegeneration and cognitive decline. Thus, Aβ deposition may serve as a trigger for damage synapses, ultimately leading to neurodegeneration and cognitive impairment.

A widely accepted theory proposed that Aβ deposition damaged synapse and neuronal loss, for which the underlying mechanisms were still controversial (47). We further explored how Aβ deposition caused the synaptic dysfunction and the events that followed. The mediation analysis revealed that in the A+ group, CSF GAP-43 mediated the association between CSF sTREM2/PGRN and CSF t-tau. The underlying mechanism linking Aβ deposition to synaptic dysfunction may involve microglia-induced neuroinflammation, with Aβ deposition acting upstream of microglia activation (48). Recent studies provided solid evidence that microglial activation increased in response to Aβ deposition. And lots of vitro studies have showed that activated microglia released soluble factors, such as TNF-, IL-6, and NO to induce synapse dysfunction (40, 49). To our knowledge, study on how Aβ deposition damages synapses in human remains limited. Herein, our findings provide evidence in human that Aβ deposition may serve as a critical initiating factor for synaptic dysfunction, and that its aggregation could damage microglia function and enhance inflammatory responses, thereby promoting synaptic injury and ultimately leading to neurodegeneration.

This study had significant advantages. As far as we know, it was the first study to reveal the impact of sTREM2/PGRN on the relationship between CSF GAP-43 and AD pathology based on population data. Moreover, we proposed a potential underlying pathway linking Aβ pathology to neurodegeneration. Nevertheless, there were also several limitations in our study. Firstly, our study lacked other synapse-related biomarkers due to sample sizes being limited in the ADNI cohort, including the presynaptic protein SNAP-25 and postsynaptic protein neurogranin. Further studies should obtain other synapse-related biomarkers to explore. Secondly, our findings focused on the cross-sectional analysis, and future studies should include more longitudinal data with additional visits and longer follow-up periods. Finally, animal experiments should be conducted to investigate the underlying mechanisms.

## 5 Conclusion

In summary, these study findings suggested that CSF GAP-43 was related to accelerated amyloid-associated neurodegeneration and cognitive decline in AD. Our data further indicated that Aβ deposition may promote microglia-mediated inflammation, leading to synaptic dysfunction. Study results provided evidence that Aβ deposition and microglia may sever as targets for future AD treatment to reduce synaptic dysfunction.

## Data Availability

Publicly available datasets were analyzed in this study. This data can be found at: www.adni-info.org.

## Footnotes

^1^ www.adni-info.org

## Group members of Alzheimer’s Disease Neuroimaging Initiative

The data used in preparation for this article were obtained from the Alzheimer’s Disease Neuroimaging Initiative (ADNI) database (adni.loni.usc.edu). As such, the investigators within the ADNI contributed to the design and implementation of ADNI and/or provided data but did not participate in the analysis or writing of this report. A complete listing of ADNI investigators can be found at: http://adni.loni.usc.edu/wp-content/uploads/how_to_apply/ADNI_Acknowledgement_List.pdf.
