# Supplementary material for: GAP-43 is associated with faster amyloid-associated neurodegeneration and cognitive decline in Alzheimer’s disease
Source: Front Neurol. 2025 Sep 26;16:1629389. doi: 10.3389/fneur.2025.1629389 (PMC12511059; doi:10.3389/fneur.2025.1629389)
Supplement: Supplementary file 1 [file Table_1.DOCX]

**Supplementary material**

**GAP-43 is associated with** **faster** **amyloid****-associated neurodegeneration** **and cognitive decline in Alzheimer's disease**

Yaxin Li ^1,#,*^, Xuanming Xu ^2,#^, Lian Tang ^3^

^1^ Department of Laboratory Medicine, Nantong First People’s Hospital and The Second Affiliated Hospital of Nantong University, Medical School of Nantong University.

^2^ Department of Clinical Laboratory, Beijing Ditan Hospital, Capital Medical University, Beijing, China.

^3^ Department of Neurology, Beijing Tiantan Hospital, Capital Medical University, Beijing, China.

^#^ These authors equally contributed to this work

^*^ Corresponding Author

**Contents**

**Fig. S1** Mediation analyses with CSF t-tau as outcomes

**Table S1** Biomarker correlations in the participants

**Table S2** Effect of biomarkers on cognitive composite measures in the participants.


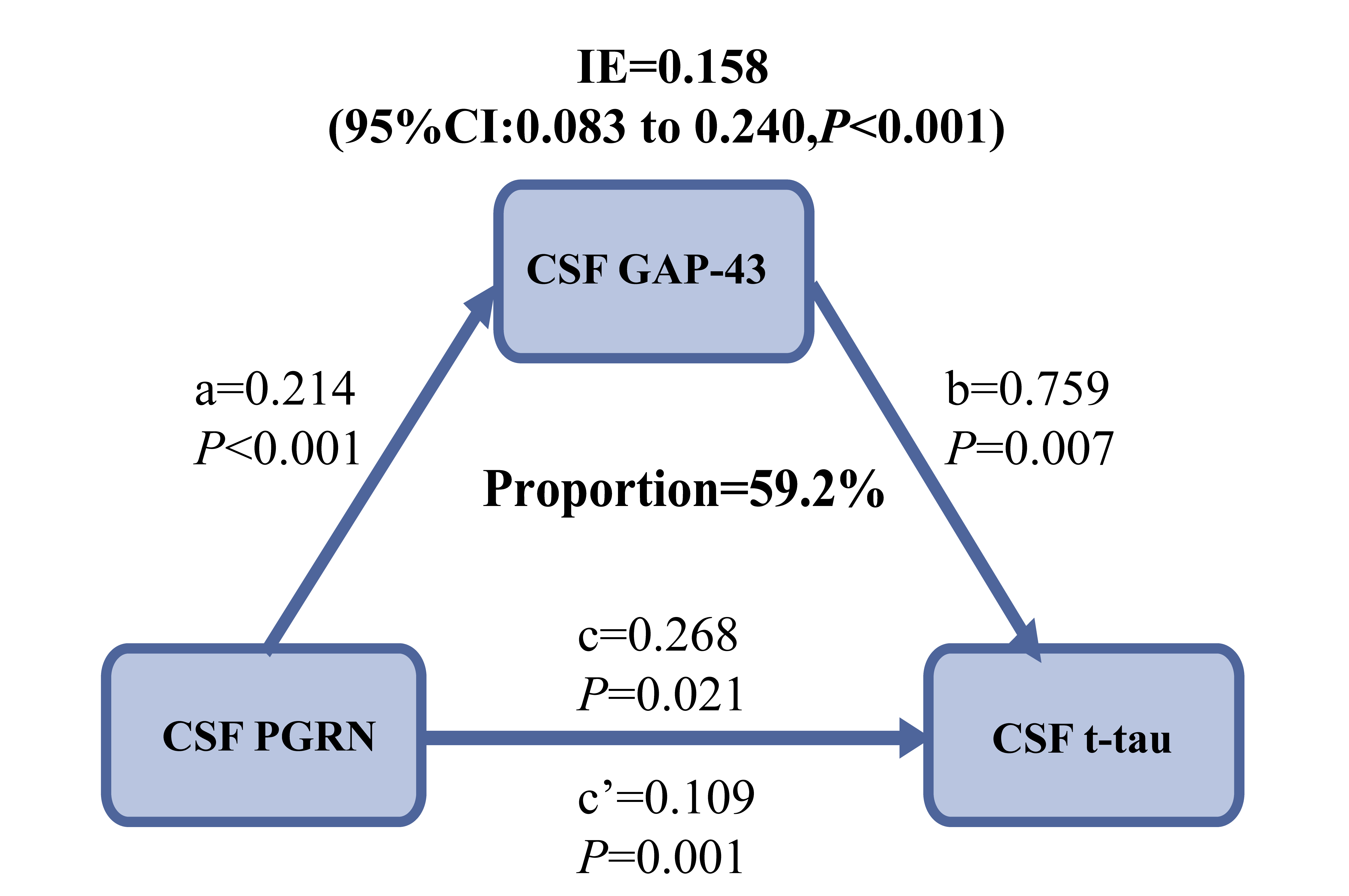


**Fig. S1** Mediation analyses with CSF t-tau as outcomes. The relationship between CSF PGRN with CSF t-tau was mediated by CSF GAP-43 in the A+ group. On the respective arrow, beta-estimates and *P*-values for each path are displayed. IE, indirect effect.

**Table S1 Biomarker correlations in the whole participants**

|  | **CSF GAP-43** | |  | **CSF sTREM2** | |  | **CSF PGRN** | |  | **Amyloid-PET** | |  | **CSF t-tau** | |
| --- | --- | --- | --- | --- | --- | --- | --- | --- | --- | --- | --- | --- | --- | --- |
|  | **β** | ***P*** |  | **β** | ***P*** |  | **β** | ***P*** |  | **β** | ***P*** |  | **β** | ***P*** |
| CSF GAP-43 | - | **-** |  | **0.441** | **<0.001** |  | **0.255** | **<0.001** |  | **0.188** | **<0.001** |  | **0.774** | **<0.001** |
| CSF sTREM2 | **0.447** | **<0.001** |  | **-** | **-** |  | **0.352** | **<0.001** |  | 0.041 | 0.244 |  | **0.450** | **<0.001** |
| CSF PGRN | **0.250** | **<0.001** |  | **0.340** | **<0.001** |  | - | **-** |  | -0.017 | 0.619 |  | **0.267** | **<0.001** |
| Amyloid-PET | **0.232** | **<0.001** |  | 0.050 | 0.243 |  | -0.022 | 0.691 |  | **-** | **-** |  | **0.414** | **<0.001** |

Adjusted *P*-values were showed in the table.

Adjusted age, gender, diagnose, education and *APOE ε4* status.

Abbreviations: CSF, cerebrospinal fluid; Aβ, amyloid-β; t-tau, total-tau; p-tau, phosphorylated-tau; GAP-43: Growth-associated protein-43; sTREM2: Soluble triggering receptor expressed on myeloid cells 2; PGRN: Progranulin.

**Table S2 Effect of biomarkers on cognitive composite measures in the participants**

|  | **Amyloid-PET** | |  | **CSF t-tau** | |  | **CSF GAP-43** | |
| --- | --- | --- | --- | --- | --- | --- | --- | --- |
|  | **β** | ***P*** |  | **β** | ***P*** |  | **β** | ***P*** |
| ADAS11 | 0.387 | **<0.001** |  | 0.264 | **<0.001** |  | 0.090 | **0.020** |
| ADAS13 | 0.382 | **<0.001** |  | 0.278 | **<0.001** |  | 0.090 | **0.010** |
| ADASQ4 | 0.306 | **<0.001** |  | 0.263 | **<0.001** |  | 0.102 | **0.006** |
| MMSE | -0.382 | **<0.001** |  | -0.295 | **<0.001** |  | -0.138 | **<0.001** |
| ADNI_MEM | -0.408 | **<0.001** |  | -0.316 | **<0.001** |  | -0.124 | **<0.001** |
| ADNI_EF | -0.304 | **<0.001** |  | -0.162 | **0.003** |  | -0.013 | 0.721 |
| CDR | 0.630 | **<0.001** |  | 0.520 | **<0.001** |  | 0.221 | **0.001** |

The normalized regression coefficients (β) and *P* values were derived from multiple linear regression.

Adjusted age, gender, education.

Abbreviations: CSF, cerebrospinal fluid; Aβ, amyloid-β; t-tau, total-tau; p-tau, phosphorylated-tau; GAP-43: Growth-associated protein-43; ADAS: Alzheimer Disease Assessment Scale; ADASQ4: ADAS delayed word recall; MMSE: Mini-Mental State Examination; ADNI-MEM: ADNI composite memory function score; ADNI-EF: ADNI composite executive function score.
